# Development of a Population Pharmacokinetic Model of Busulfan in Children and Evaluation of Different Sampling Schedules for Precision Dosing

**DOI:** 10.3390/pharmaceutics14030647

**Published:** 2022-03-15

**Authors:** Efthymios Neroutsos, Ricardo Nalda-Molina, Anna Paisiou, Kalliopi Zisaki, Evgenios Goussetis, Alexandros Spyridonidis, Vasiliki Kitra, Stelios Grafakos, Georgia Valsami, Aristides Dokoumetzidis

**Affiliations:** 1 Laboratory of Biopharmaceutics & Pharmacokinetics, Department of Pharmacy, School of Health Sciences, National & Kapodistrian University of Athens, 15784 Athens, Greece; enerouts@pharm.uoa.gr (E.N.); valsami@pharm.uoa.gr (G.V.); 2 Department of Engineering, School of Pharmacy, Miguel Hernández University, 03550 San Juan de Alicante, Spain; jnalda@umh.es; 3 Alicante Institute for Health and Biomedical Research (ISABIAL-FISABIO Foundation), 03010 Alicante, Spain; 4 Bone Marrow Transplant Unit, “Agia Sophia” Children’s Hospital of Athens, 15127 Athens, Greece; salamanca.creta@yahoo.gr (A.P.); kalliopizisaki@gmail.com (K.Z.); evgoussetis@gmail.com (E.G.); vkitra@hotmail.co.uk (V.K.); s.graphakos@paidon-agiasofia.gr (S.G.); 5 Department of Internal Medicine, Bone Marrow Transplantation Unit, University Hospital of Patras, 26504 Patras, Greece; spyridonidis@upatras.gr

**Keywords:** busulfan, paediatric, acute myelogenous leukemia, hematopoietic stem cell transplantation, pharmacometrics, model-informed precision dosing

## Abstract

We develop a population pharmacokinetic model to describe Busulfan pharmacokinetics in paediatric patients and investigate by simulations the impact of various sampling schedules on the calculation of AUC. Seventy-six children had 2 h infusions every 6 h. A two-compartment linear model was found to adequately describe the data. A lag-time was introduced to account for the delay of the administration of the drug through the infusion pump. The mean values of clearance, central volume of distribution, intercompartmental clearance, and peripheral volume of distribution were 10.7 L/h, 39.5 L, 4.68 L/h and 17.5 L, respectively, normalized for a Body Weight (BW) of 70 kg. BW was found to explain a portion of variability with an allometric relationship and fixed exponents of 0.75 on clearance parameters and 1 on volumes. Interindividual variability for clearance and volume of distribution was found to be 28% and 41%, respectively, and interoccasion variability for clearance was found to be 11%. Three sampling schedules were assessed by simulations for bias and imprecision to calculate AUC by a non-compartmental and a model-based method. The latter was found to be superior in all cases, while the non-compartmental was unbiased only in sampling up to 12 h corresponding to a once-daily dosing regimen.

## 1. Introduction

Busulfan (BU) is widely used as a conditioning regimen before hematopoietic stem cell transplantation (HSCT) in patients with chronic myelocytic leukemia and other haematological malignancies such as non-Hodgkin lymphomas, immune deficiencies, and thalassemia [1,2,3,4,5,6,7,8,9]. In 1983, Santos et al. [3] used oral BU at a dose of 1 mg/kg every six hours for 16 doses in combination with cyclophosphamide as a preparative pattern for patients with acute myelogenous leukemia who were undergoing HSCT. Although oral BU presented high inter- and intra-individual variability [4] and a relatively narrow therapeutic range between 900 and 1500 μM × min, expressed in AUC units (area under the plasma concentration–time curve), it gradually replaced total body irradiation due to the physical and mental retardation, caused by the latter, especially in children. Anderson et al. [10] observed that intravenous (iv) BU administration (Busilvex^®^ Pierre Fabre Medicament, Boulogne, France) at a dose of 0.8 mg/kg in a 2 h infusion every 6 h for 16 consecutive doses was safer than the orally administered BU. Russell et al. [11] optimized the dose of intravenous-administered BU at 3.2 mg/kg in a 3 h infusion once daily for four consecutive days, which proved to be the safest dosage scheme. However, even intravenous-administered BU presents high variability and therefore its administration must be individualized based on blood concentration measurements.

BU pharmacokinetics varies in children between 3 and 7 years, while the optimum therapeutic range (TR) has not yet been specified due to the limited number of studies on the paediatric population [12,13,14,15,16,17]. It is evident, however, that a higher plasma AUC is associated with a high-risk of regimen-related toxicity and, in particular, veno-occlusive disease (VOD) of the liver [12,14,16], while low busulfan plasma levels are associated with a higher risk of graft rejection [9] and leukemia relapse [12]. Accordingly, BU TDM after iv administration in children is recommended according to the drug’s Summary of Product Characteristics (SmPC) [18].

In paediatric patients, low dose BU administration is very often dependent on patient’s age and body weight. For this purpose, syringe pumps are used for the micro-infusion of the drug [19]. However, this procedure results in relative high lag-time before drug entrance into the blood circulation [20]. In addition, variable infusion rates are observed in this population based on the pump program, the changes in the hydrostatic pressure as well as the physicochemical properties of the infused drugs, resulting in significant delay of blood concentration steady state achievement [21,22,23]. The resulting lag-time varies between 10 and 40 min and should be considered in clinical practice to avoid dosing errors, especially in paediatric patients for whom low infusion rates are usual [21,22,23].

According to BU SmPC [18], dose individualization is recommended in paediatric patients and is based on the calculation of AUC using the log-linear trapezoidal rule. However, lag-time due to syringe-pump application may influence the calculation of AUC if it is not considered, resulting in false dose corrections, especially for AUC values close to the TR limits [18]. The effect of lag-time in the calculation of AUC in BU TDM has been studied in [24].

In the present study, we aimed to analyse concentration–time profiles after BU administration to paediatric patients from the study in [24] and develop a population pharmacokinetic model to describe BU pharmacokinetics in the paediatric population, incorporating the lag-time on BU entrance to blood circulation during administration to paediatric patients using a syringe-pump, based on the patient’s body weight. Furthermore, we investigate the impact of various sampling schedules on the calculation of AUC through by simulations based on the developed model.

## 2. Materials and Methods

### 2.1. Study Population and Blood Sampling

The clinical study was performed at the Bone Marrow Transplantation Unit of the “Agia Sofia” Children’s Hospital of Athens, between July 2014 and January 2017. It was a prospective, dose individualization clinical study to paediatric patients undergoing bone marrow transplantation after preparative chemotherapy treatment, including BU iv. Patients with one of the following diseases were included in the study: Adrenoleukodystrophy, acute lymphoblastic leukemia, T-cell acute lymphoblastic leukemia, acute myeloid leukemia, B-cell acute myeloid leukemia, anaemia Blackfan–Diamond, sarcoma Ewing, myelodysplastic syndrome, neuroblastoma stage IV, non-Hodgkin lymphoma, thalassaemia, Wiskott–Aldrich syndrome.

Seventy-six paediatric patients were included in the study. Their demographic data are shown in Table 1. All patients were administrated BU with 2 h iv infusion every 6 h, for 16 doses of 0.8–1.2 mg/kg, depending on patient’s BW, according to the recommendations described in the SmPC of the administered product (Busilvex^®^) [18]. The characteristics of the patients’ population for each BU dosage category are given in Table 2, together with the administered dose. For each patient, age, height, weight, gender, and creatinine clearance (expressed as CKPD-EPI) were recorded, as well all other co-administered drugs and possible residual diseases. All patients (or their guardian) gave informed consent, and the study was conducted in accordance with the Declaration of Helsinki and approved by the Ethics Committee of the Hospital (Appr. Number 22050, date of approval: 29 September 2014).

For each patient, blood was collected on the first day, and most patients also had blood collected on the second day, at the following times: before the BU infusion and immediately after, 30 min, 2 h, and 4 h after the completion of first BU administration, i.e., at nominal times: 0, 2, 2.5, 4 and 6 h. A total of 596 blood samples were collected and centrifuged at 3000× *g* for 10 min and stored at −80 °C until analysis by recently optimized and validated HPLC-PDA bioassay method for the quantitation of BU in biologic fluids [24].

To determine the possible lag-time occurring during infusion, we simulated in vitro the infusion administration procedure with the BW stratification used for the selection of the dose [18]. The details of the in vitro investigation are published elsewhere [24].

### 2.2. Population Pharmacokinetic Modeling

Non-Linear Mixed Effect Modeling (NLME), applying the first-order conditional (FOCE) method implemented in NONMEM^®^ 7.3 software package (ICON, Hanover, MD, USA), was used to develop the pharmacokinetic model to describe the measured BU plasma concentrations versus time data and to conduct model-based simulations.

One and two compartment structural models with and without infusion lag-time (T_lag_) were tested and parameterized as either CL and V_1_ (ADVAN 1 TRANS 2 routine), or CL, V_1_, Q, and V_2_ (ADVAN 3 TRANS 4 routine), respectively, where CL is the clearance (L/h), V_1_ is the central volume of distribution (L), Q is the inter-compartmental clearance (L/h), and V_2_ is the volume of the distribution of the peripheral compartment (L). The infusion lag-time was added using the NONMEM parameter ALAG1, which delays the administration. The lag-time for each patient came from Table 2 and was included in the dataset. The additive, the proportional, and the combined residual error models were tested to describe residual variability. Furthermore, inter-individual variability (IIV) and inter-occasion variability (IOV) were considered for parameters CL, V_1_, Q and V_2_. Age, BW, body surface area (BSA), and creatinine clearance (calculated using CKD-EPI equation [25]) were tested as covariates on the parameters of the final model.

### 2.3. Model Selection Criteria

Model comparison between competing models for the covariates to be included in the final model was performed using the likelihood ratio test. According to the test, the inclusion of a covariate involving one additional parameter is considered statistically significant, at a significance level α = 0.05, when the objective function value reduces by at least 3.84 units (ΔOFV > 3.84). Furthermore, besides statistical significance, a covariate is expected to reduce the stochastic variability associated with the specific parameter. Improvement of the diagnostic plots and soundness of the physical interpretation of the covariate was also assessed.

### 2.4. Pharmacokinetic Model Qualification

A nonparametric bootstrap analysis was performed as internal model validation using the software Perl Speaks Nonmem (PsN) [26]. The bootstrap analysis tests the robustness of the model and its sensitivity to specific individual patients and is also an alternative way to calculate the uncertainty of the parameter estimates. The analysis consists of constructing 1000 random datasets by replacement of individuals from the original dataset. The model is fitted to each of these datasets and descriptive statistics are calculated for the set parameter estimates from these runs. The final model’s parameter estimates are considered unbiased if they lie within the 95% Confidence Intervals (CI) of the bootstrap estimates. The Standard Deviations of the bootstrap estimates represent uncertainty.

In addition, a prediction corrected visual predictive check (pcVPC) was performed using the final model parameter estimates to simulate the population pharmacokinetic profile of BU in plasma. The pcVPC plot was generated by PsN [26] and was presented with respect to the time after dose (TAD). Unlike ordinary VPC, the pcVPC normalizes all predictions and observations using typical predicted values and therefore is more appropriate for heterogenous datasets, eliminating the need for stratified comparisons. Heterogenous datasets may include paediatric datasets with high impact covariates, datasets with different dosing regimens, or even different administration occasions.

### 2.5. Evaluation of Model Performance for Dose Individualization

According to the recommendation for the dose individualization of iv-administered BU in the paediatric population, the AUC is the PK parameter of choice [18]. In our study the Empirical Bayesian Estimate (EBEs) of CL calculated using only the samples of the first day for each patient were used to calculate AUC values to be used for dose individualization, denoted as AUC_bayes_. For comparison, AUC values were calculated using the trapezoidal rule, taking into account the infusion lag-time (T_lag_) with the methodology described in [24], denoted as AUC_trap_. The percentage of patients within the TR (TR, 900–1500 μM × min) was calculated using the AUC values derived from both methodologies.

To evaluate the performance of AUC_bayes_ with respect to AUC_trap_, stochastic simulations of the final PopPK model were performed in 1000 patients. The covariates were randomly selected from the original dataset. Three sampling schedules were assessed in the simulations: Schedule 1 (original): 2.5, 3, 4 and 6 h; Schedule 2 (reduced): 2.5, 4 and 6 h; Schedule 3 (long): 3, 6, 9 and 12 h. The latter corresponds to a once-daily dosing regimen which includes a 3 h infusion with a dose 4 times higher the qid dose [11,15]. Due to the high dose of this regimen, the associated lag-time is negligible. Bias and Imprecision were used to compare the AUC_bayes_ and the AUC_trap_ with the true AUC, denoted as AUC_true_ (the one calculated with the true CL obtained in the simulation). Bias and Imprecision were assessed by the mean precision error (MPE, Equation (1)) and the root mean square prediction error (RMSPE, Equation (2)). The closer to zero Bias and Imprecision are, the more accurate and precise the method is.

(1) $$\mathrm{MPE}=\frac{\sum \left({\mathrm{AUC}}_{\mathrm{true}}-{\mathrm{AUC}}_{\mathrm{calc}}\right)}{n}$$

(2) $$\mathrm{RMSPE}=\sqrt{\frac{\sum {\left({\mathrm{AUC}}_{\mathrm{true}}-{\mathrm{AUC}}_{\mathrm{calc}}\right)}^{2}}{n}}$$

where AUC_calc_ represents either the AUC_bayes_ or the AUC_trap_, and *n* is the number of patients.

## 3. Results

### 3.1. Development of the Structural and Base PopPK Model

Based on literature data, BU is characterised by linear pharmacokinetics, described by either one [8] or two compartment distribution models [16] with first order elimination. Accordingly, both one and two compartment PK models were tested with and without infusion lag-time. The two-compartment distribution model including the lag-time predefined as a covariate, and first order elimination was found to better describe the observed concentration vs. time data, resulting to a lower OFV. The residual variability was better described by the combined (proportional + additive) error model. IIV was estimated for CL and V_1_, while correlation between CL and V_1_ for IIV was also included in the model. The incorporation of IOV in cases of blood samples collected at different administration occasions further decreased the OFV. Diagnostic plots (observed vs. predicted plasma concentrations plots, residual error plots, NPDE plots) showing the goodness of fit for the base PopPK model are given in Figure S1 in the Supplementary Materials, while, in Table S1 in the Supplementary Materials, the estimated PK parameter values are presented together with the results from the nonparametric bootstrap analysis.

### 3.2. Development of the Final Covariate Model

For the development of the covariate model, height, BW, post menstrual age (PMA), creatinine clearance, and BSA were checked for their possible effect as covariates of the base PopPK model.

BW introduced as a power function for both CL and V_1_ improved the fit, significantly dropping the OFV value from −1591.275 to −1713.474. However, the estimated values of the exponents were 0.808 and 1.04 for CL and V_1_, respectively, and a similar OFV value (−1712.152) was achieved by the allometric model of fixed exponents 0.75 and 1 on CL and V_1_, respectively, which was preferred. Adding the BW as a covariate to Q and V_2_ using the fixed exponents of 0.75 and 1 reduced the OFV further. The random effects model was revisited and the IOV on V_1_ had a high SE while the additive residual error had a relatively small value as well as an elevated SE, and both were dropped from the final model resulting in a final OFV of −1719.552.

**Figure 1:**
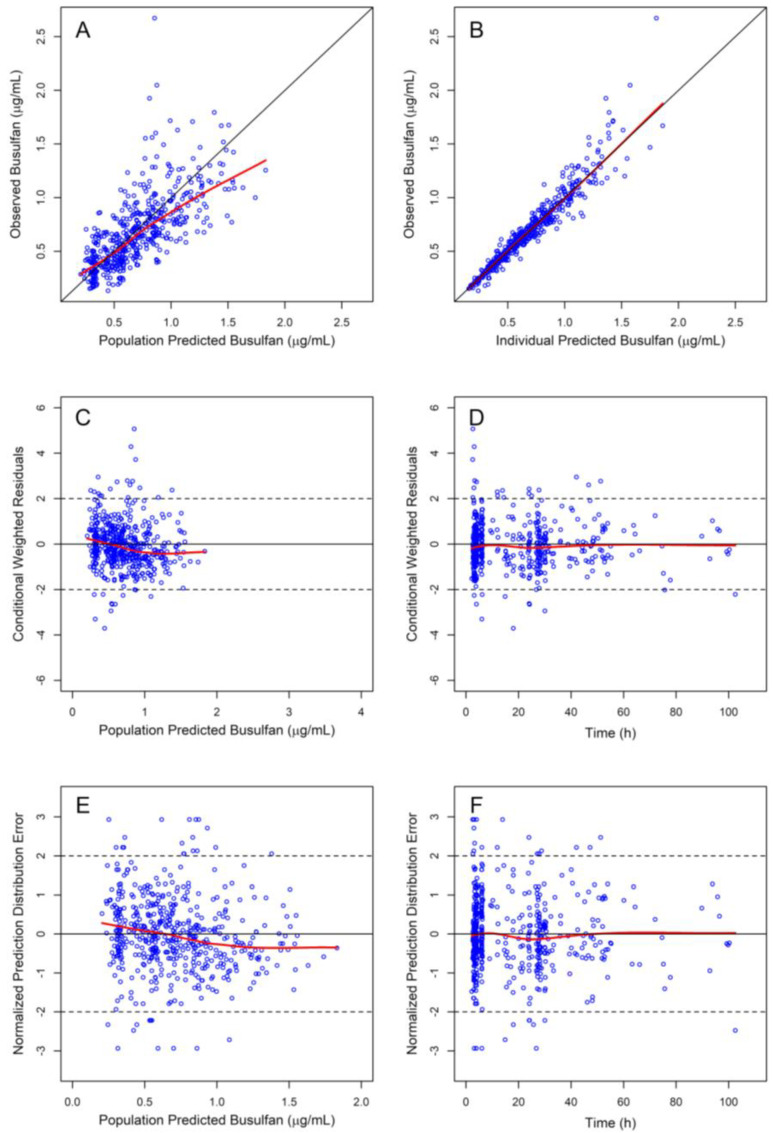
Diagnostic plots for the final PopPK model. Observed vs. population predicted plasma concentrations (**A**) and individual predicted plasma concentrations (**B**) plots (black and red lines represent the identity and cubic spline smooth lines, respectively). Conditional weighted residuals vs. population predicted plasma concentrations (**C**) and vs. TIME (**D**) (solid line y = 0, dashed lines y = 2 and y = −2). Normalized Prediction Distribution Error vs. population predicted plasma concentrations (**E**) and vs. Time (**F**).

**Figure 2:**
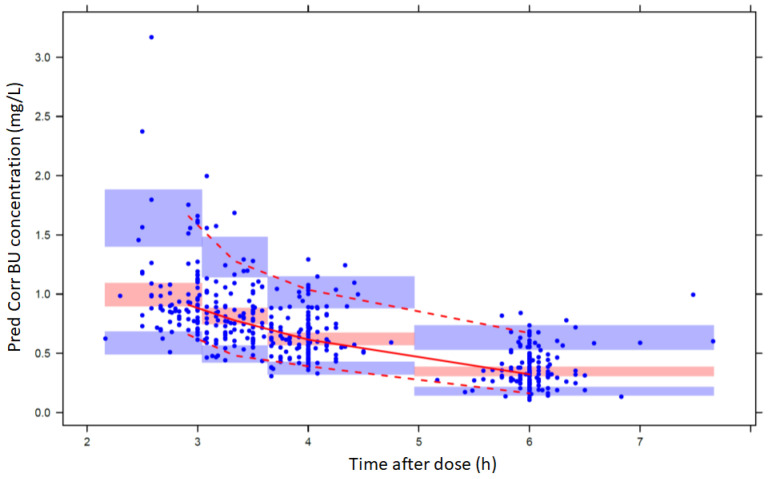
pcVPC for the final model. Red lines are the 5, 50 and 95% prediction intervals. The shaded areas represent the 95% confidence interval for the estimation.

The bias observed in the diagnostic plots was also corrected with the addition of the covariate in the model. Furthermore, age was tested as a covariate in the model in the form of a Hill equation to describe the maturation of CL [27]. Overall, introduction of age did not improve the model significantly, probably due to the small number of children (four) below 2 years of age included in the dataset. BSA and creatinine clearance were also tested as covariates but did not improve the fit of the model to the observed data. The equations of the final model that describe the pharmacokinetic parameters are the following: $CL_{i}=CL_{t}\cdot {\left(BW/70\right)}^{0.75}$, $V_{1,t}=V_{1,t}\cdot \left(BW/70\right)$, $V_{2,t}=V_{2,t}\cdot \left(BW/70\right)$ and $Q_{i}=Q_{t}\cdot {\left(BW/70\right)}^{0.75}$, where, *i* subscript denotes the *i*th patient and *t* subscript denotes the typical value corresponding to a 70 kg patient. Supplementary Materials contains the NONMEM script of the final model. In Table 3, the estimated parameters’ values, together with the results from the nonparametric bootstrap analysis, are reported for the final model. The NONMEM parameter estimates lie within the 95% CIs of the bootstrap analysis, indicating that they are unbiased; furthermore, the %CV of the bootstrap analysis, as well as the NONMEM Standard Errors, are reasonable and comparable to each other. The final model is supported by the goodness of fit plots (Figure 1) and the pcVPC, expressed in terms of TAD (Figure 2). *CL* and *V*_1_ η-Shrinkage for the final model were 6.7% and 11.0%, respectively, while ε-shrinkage was 16.0%. The relatively low value of the shrinkage for CL is a key for the unbiased estimation of AUC used in the dose individualization of BU. The mean values of *CL* and *V*_1_ were 10.7 L/h and 39.5 L, for 70 kg body weight, respectively. The differences between these values and the values in the base model are due to the centring of the covariate at 70 kg, while the mean BW in the population is 30.6 kg. Considering the range of weights in the studied population (7.38–104 kg), the values for the population *CL* and *V*_1_ range from 1.98–14.4 L/h and 4.16–58.7 L, respectively. The mean terminal half-life of all patients is about 4 h, with a CV of 22%, while patients with a *BW* of 10 kg have a half-life of about 3 h and patients with a BW above 50 kg have higher half-life values near 5 h.

### 3.3. BU Dose Individualization

The recommended procedure in the Busilvex^®^ label is by using AUC_trap_ as the pharmacokinetic target. Therefore, it was considered as the reference to be compared with. Considering the AUC_trap_, the percentage of patients below, within and above the TR are 50.7%, 41.1%, and 8.2%, respectively. On the other hand, when the AUC_bayes_ was used, the percentage of patients below, within and above the TR, are 19%, 58.9% and 21.9%, respectively. It is clear that the trapezoidal method tends to obtain lower values of AUC than the Bayesian method.

### 3.4. Evaluation of Model Performance for Dose Individualization

**Figure 3:**
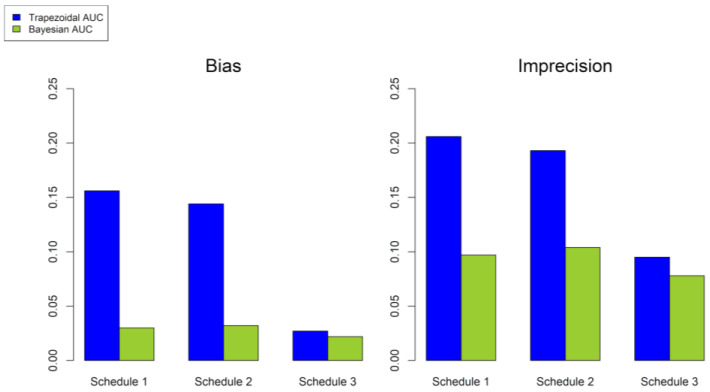
Bias and imprecision of the AUC calculated through the trapezoidal and the Bayesian approximation. Sampling times are as follows: Schedule 1: 2.5, 3, 4 and 6 h; Schedule 2: 2.5, 4 and 6 h; Schedule 3: 3, 6, 9 and 12 h.

The bias and imprecision obtained after evaluating the stochastic simulations are shown in Figure 3. The AUC_bayes_ performed much better in terms of bias and imprecision than the AUC_trap_ in Schedules 1 and 2. However, the AUC_trap_ in Schedule 3 had an improved performance, similar to AUC_bayes_. The terminal slopes used to obtain the area from the time of the last concentration to infinity in the AUC_trap_ were calculated using the last three sampling times of each simulated patient, e.g., Schedule 1: 3, 4 and 6 h; Schedule 2: 2.5, 4 and 6 h; Schedule 3: 6, 9 and 12 h

## 4. Discussion

In the present study, plasma concentration vs. time data were used to develop a PopPK model for iv BU in the paediatric population. A two-compartment distribution model with first order elimination was found to adequately describe BU pharmacokinetics while BW was found to explain a portion of variability with an allometric relationship. While, in the past, BU has been modelled mainly by one-compartment models, our findings of the presence of a second compartment not only confirm other recent studies [16], but also have important clinical implications, as shown in the simulation study of the previous section and discussed further down. This is despite the fact that the improvement in goodness of fit between the one and the two compartment models was clear but still moderate. In Bartelink et al. (2016) [16], the authors also developed a two-compartment model to describe paediatric iv busulfan data, while clearance was found to be 3.51 L/h for their typical patient of 15.3 kg, and the central volume of the distribution was found to be 11.1 L. For a patient of 15.3 kg the respective parameter values of our model are 3.4 L/h for CL and 8.64 L for V_1_.

In addition, the infusion lag-time (T_lag_) that is observed mainly when small volumes of BU solution are administered by iv infusion using a pump-syringe, which has been simulated in vitro [24], was incorporated in the developed PopPK model. Inclusion of the T_lag_ improved the model and reduced the objective function. T_lag_ values for each of the BU dose categories described in the SmPC of the administered product were determined and were found to decrease by increasing the infusion volume (the dose), reaching a minimum of less than 10 min for BW > 34 kg. Small infusion volumes, which are comparable to the line’s capacity, can impact the correct estimation of AUC in small children, while these become negligible in older children. The impact of lag-time can be significant for the accuracy of the AUC estimate if ignored when the dose is very small, as mentioned in [24]. Note that our study involved few children of the low weight band, most affected by the lag-time.

**Figure 4:**
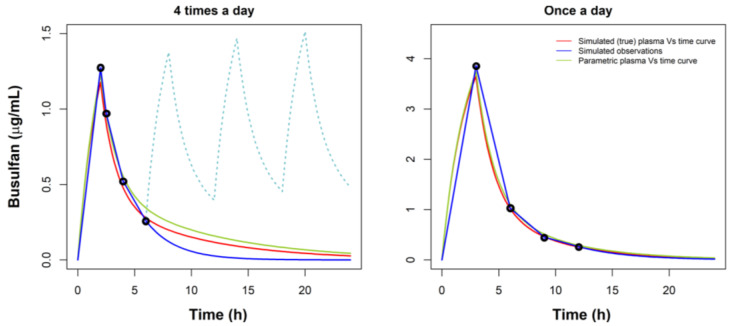
Two representative simulated patients with 3 different approximations in the AUC calculations. Red line represents the true AUC, considering the true PK parameters obtained in the simulations; blue line represents the trapezoidal AUC, considering the simulated observations; green line represents the Bayesian AUC, considering the PK parameters obtained after the Bayesian approach.

The main clinical conclusion of the study is the impact of the second compartment to the accurate calculation of the AUC for the purpose of dose adjustment. Indeed, as presented in the results section, simulations with the model and calculation of the AUC by the trapezoidal method and by the Bayesian estimation of CL, in various scenarios, reveal that the calculation of the AUC by the trapezoidal method, using sampling up to 6 h, may lead to significant systematic underestimation of the AUC, mainly due to poor estimation of the terminal slope for the extrapolation of AUC to infinity. This is due to the sampling times used to calculate the slope, including part of the fast distribution region of the curve and not the terminal part, therefore the slope tends to be overestimated. Moreover, the underestimation of AUC had a pronounced impact on the potential decisions made for the allocation of each patient within or outside the TR. In the same sampling scenarios, a model-based approach using the EBE estimates of CL leads to more accurate estimates. On the other hand, a once-daily dosing regimen (Schedule 3) [11,15], apart from the advantage of being more patient-friendly, involving only 3 h of infusion as opposed to the total of 8 h of the four-times-daily Schedule (2 h × 4), allows to take later samples (e.g., up to 12 h) and describes the terminal part of the curve in better detail, so the slope is calculated with a higher accuracy, which leads to better estimates of AUC when using the trapezoidal method. Obviously, using the model-based method with the once-daily sampling scheme gives the best overall results. The once-daily regimen also has the advantage of eliminating the impact of lag-time due to the four-times higher dose used. Figure 4 illustrates the impact of the slope calculation in two representative simulated patients, corresponding to the three different schedules.

## 5. Conclusions

Our study confirms previous studies, highlighting that the need of dose adjustment of IV busulfan is necessary for paediatric patients undergoing HSCT. Furthermore, the pivotal role of a model-based approach using pharmacometric models is confirmed and is preferable to the classic NCA approach even for iv administration. The model which best describes the data in our study is two-compartmental, and we found that the impact of the presence of the second compartment is very significant in the accuracy of the calculation of AUC for the purpose of dose adjustment due to the misspecification of the terminal slope when data sampling stops at 6 h, as is often the case. On the other hand, a once-daily dosing regimen allowing sampling up to later times gave better results both with NCA and the model-based approaches. Finally, the effect of lag-time has been found to be important in small doses and can bias results if ignored.

## Figures and Tables

**Table 1:** Demographic characteristics of the patients.

Parameter	Mean/Number	SD	Range
Number of patients	76		
Male patients	49		
Age (years)	7.6	5.1	0.5–19
Body Weight (kg)	30.6	21.6	7.38–104
Height (cm)	121.8	35.2	0.71–207
BSA (m^2^)	0.99	0.49	0.09–2.44
Sr Cr (mg/dL)	0.36	0.15	0.10–0.90
CKPD-EPI (mL/min/1.73 m^2^)	197	41	107–346

**Table 2:** Dosing chart including weight and age distribution for each category.

BW Category (kg)	Dose (mg/kg)	Lag-Time (min)	Number of Patients (Ν)	Median BW, kg (Range)	Median Age, Years (Range)
<9	1	40	4	8.02 (7.4–8.7)	0.7 (0.58–0.75)
9–16	1.2	40	20	12.31 (9.2–15.0)	3.1 (0.5–7.0)
16–23	1.1	35/25	13	19.35 (16.0–66.7)	5.23 (3.0–7.0)
23–34	0.95	20	16	28.7 (25.0–34.0)	7.9 (5.0–12.0)
>34	0.8	10/5	23	58.1 (34.5–104.0)	13.9 (6.0–19.0)

**Table 3:** Parameter estimates using the final covariate PopPK model.

Parameter	ΝOΝΜΕΜ Estimation			Bootstrap Analysis			
	Estimate	SE	RSE%	Mean	SD	CV%	CI (2.5–97.5%)
CL (L/h)	10.7	0.431	4.05%	10.7	0.430	4.04%	9.79–1.47
V_1_ (L)	39.5	2.70	6.84%	39.1	2.66	6.79%	34.0–44.3
Q (L/h)	4.68	0.712	15.2%	4.78	0.701	14.7%	3.65–6.30
V_2_ (L)	17.5	3.00	17.2%	17.5	3.06	17.5%	12.4–24.4
CL IIV	0.284	0.0147	5.18%	0.282	0.0259	9.16%	0.231–0.335
V_1_ IIV	0.409	0.058	14.2%	0.412	0.0728	17.7%	0.267–0.554
Cor. CL-V_1_	0.679	0.025	3.68%	0.680	0.0807	11.9%	0.495–0.813
CL IOV	0.105	0.00259	2.47%	0.103	0.0125	12.1%	0.078–0.127
Prop. RE *	0.126	0.00208	1.65%	0.125	0.0082	6.56%	0.109–0.141

* RE = residual error.

## Data Availability

The data presented in this study are available on request from the corresponding author.

## Supplementary Materials

The following supporting information can be downloaded at: https://www.mdpi.com/article/10.3390/pharmaceutics14030647/s1, Figure S1: Diagnostic plots for the Base PopPK model; Table S1: Parameter estimates using the base PopPK model; NONMEM Control script.
