# Supplementary material for: Development of a Population Pharmacokinetic Model of Busulfan in Children and Evaluation of Different Sampling Schedules for Precision Dosing
Source: Pharmaceutics. 2022 Mar 15;14(3):647. doi: 10.3390/pharmaceutics14030647 (PMC8948694; doi:10.3390/pharmaceutics14030647)
Supplement: Supplementary file 1 [file pharmaceutics-14-00647-s001.zip › pharmaceutics-1603221-supplementary.pdf]

pharmaceutics

MDPI

Article

Development of a population pharmacokinetic model of busulfan in children and evaluation of different sampling schedules for precision dosing

Efthymios Neroutsos, Ricardo Nalda-Molina, Anna Paisiou, Kalliopi Zisaki, Evgenios Goussetis, Alexandros Spyridonidis, Vasiliki Kitra, Stelios Grafakos, Georgia Valsami and Aristides Dokoumetzidis

Table S1. Parameter estimates using the base PopPK model.

	NONMEM Estimation			Bootstrap Analysis			
**PK parameter**	**Estimate**	**SE**	**RSE%**	**Mean**	**SD**	**CV%**	**CI (2.5%–97.5%)**
CL (L/h)	4.99	0.348	6.97	4.94	0.365	7.38	4.35-5.69
V_1 (L)	13.9	1.37	9.86	13.98	1.48	10.57	11.3-17.18
V_2 (L)	3.69	0.466	12.63	3.88	0.71	18.24	2.95-5.88
Q (L/h)	1.55	0.130	8.39	1.51	0.16	10.56	1.19-1.80
CL IIV	0.60	0.0358	5.97	59.2	0.03	5.68	0.53-0.65
V_1 IIV	0.67	0.0703	10.49	77.5	0.08	9.90	0.63-0.91
Cor. CL-V_1	0.94	0.0268	2.85	0.94	0.03	2.68	0.89-0.99
CL IOV	0.11	0.0150	13.64	11.5	0.01	12.99	0.09-0.14
V_1 IOV	0.18	0.0648	36.00	17.9	0.06	32.77	0.07-0.29
Prop. RE*	0.114	0.00977	8.57	0.11	11.2	9.73	0.08-0.13
Add. RE*	0.0229	0.011	48.03	0.03	0.01	35.52	0.01-0.04

*RE= residual error.

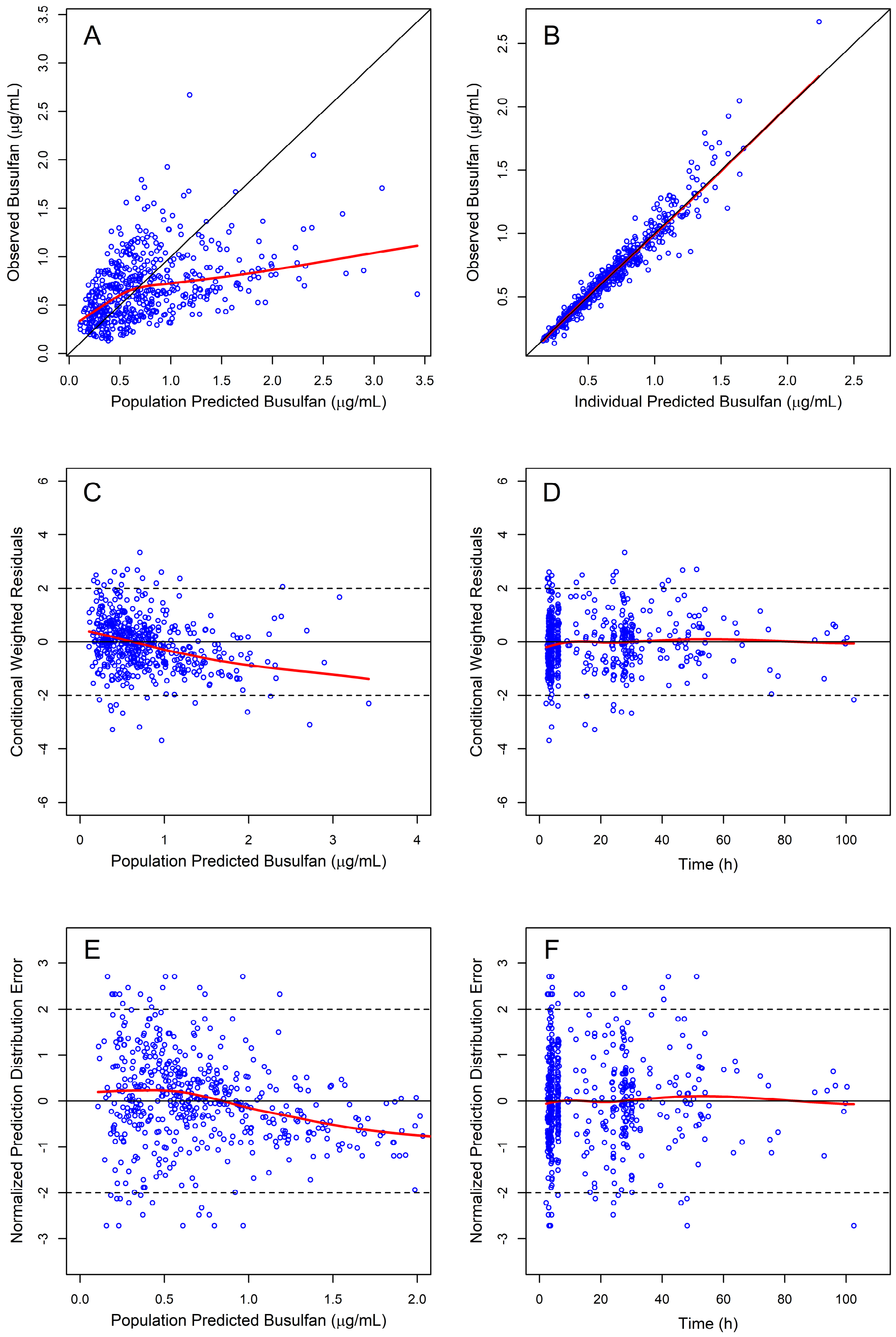

Figure S1. Diagnostic plots for the Base PopPK model. Observed vs population predicted plasma concentrations (**A**) and individual predicted plasma concentrations (**B**) plots (black and red lines represent the identity and cubic spline smooth lines, respectively). Conditional weighted residuals vs population predicted plasma concentrations (**C**) and vs TIME (**D**) (solid line y = 0, dashed lines y = 2 and y = -2). Normalized Prediction Distribution Error vs population predicted plasma concentrations (**E**) and vs Time (**F**).

NONMEM Control script

```
$PROBLEM    Busulfan Final Model
$INPUT    ID TIME DV EVID AMT TLAG RATE BW MDV OCC

$DATA    Busulfan_Data.csv    IGNORE=#
$SUBROUTINES    ADVAN3 TRANS4

$PK
   IF(NEWIND.LT.2) THEN
      IFL=0
      TAD=0.0
   ENDIF

   IF(EVID.EQ.1.OR.EVID.EQ.4) THEN
      TDOS=TIME
      TAD=0.0
      IFL=1
   ENDIF

   IF(IFL.EQ.1.AND.EVID.NE.1.AND.EVID.NE.4)TAD=TIME-TDOS

   OC1 = 0
   OC2 = 0
   OC3 = 0
   OC4 = 0
   OC5 = 0
      IF (OCC.EQ.1) OC1=1
      IF (OCC.EQ.2) OC2=1
      IF (OCC.EQ.3) OC3=1
      IF (OCC.EQ.4) OC4=1
   OCC1 = OC1*ETA(3)    + OC2*ETA(4)    + OC3*ETA(5)

      TCL=THETA(1)
      A=(BW/70)**THETA(5)
   CL=TCL* A* EXP(ETA(1))* EXP(OCC1)
      TV1=THETA(2)*(BW/70)**THETA(6)
   V1=TV1*EXP(ETA(2))
   V2=THETA(3)*(BW/70)
   Q=THETA(4)*(BW/70)**THETA(5)

   ALAG1=Tlag
   S1=V1

$THETA       (0 11)
         (0 41)
         (0 3.77)
         (0 1.49)
         (0 0.75) FIX
         (0 1) FIX

$OMEGA    BLOCK(2)    0.0697 0.0662 0.106

$OMEGA BLOCK(1) 0.0125
$OMEGA BLOCK(1) SAME
$OMEGA BLOCK(1) SAME
```

```
$SIGMA 0.0136

$ERROR
    IPRED=F
    Y=F+F*EPS(1)
    IRES=DV-IPRED
    IWRES=IRES/IPRED

$ESTIMATION METHOD=1 INTERACTION MAXEVAL=9999 PRINT=2

$TABLE ID TIME AMT IPRED EVID MDV IWRES CWRES NPDE NOPRINT ONEHEADER
FILE=sdtab1
$TABLE ID CL V1 V2 Q NOPRINT ONEHEADER FILE=patab1
```
